# Exploring the direct and indirect effects of elite influence on public opinion

**DOI:** 10.1371/journal.pone.0257335

**Published:** 2021-11-19

**Authors:** Lauren Ratliff Santoro, Elias Assaf, Robert M. Bond, Skyler J. Cranmer, Eloise E. Kaizar, David J. Sivakoff

**Affiliations:** 1 Department of Political Science, The University of Texas at Dallas, Dallas, Texas, United States of America; 2 Department of Political Science, The Ohio State University, Columbus, Ohio, United States of America; 3 School of Communication, The Ohio State University, Columbus, Ohio, United States of America; 4 Department of Statistics, The Ohio State University, Columbus, Ohio, United States of America; 5 Department of Mathematics, The Ohio State University, Columbus, Ohio, United States of America; University of Granada: Universidad de Granada, SPAIN

## Abstract

Political elites both respond to public opinion and influence it. Elite policy messages can shape individual policy attitudes, but the extent to which they do is difficult to measure in a dynamic information environment. Furthermore, policy messages are not absorbed in isolation, but spread through the social networks in which individuals are embedded, and their effects must be evaluated in light of how they spread across social environments. Using a sample of 358 participants across thirty student organizations at a large Midwestern research university, we experimentally investigate how real social groups consume and share elite information when evaluating a relatively unfamiliar policy area. We find a significant, direct effect of elite policy messages on individuals’ policy attitudes. However, we find no evidence that policy attitudes are impacted indirectly by elite messages filtered through individuals’ social networks. Results illustrate the power of elite influence over public opinion.

## Introduction

Political elites do not simply “pander” to public opinion, they seek to shape and influence it [1, 2]. Elite messaging about policy frequently targets the public. For example, elites use media, campaign events, and press releases, among other avenues, to make direct appeals about policy to members of the public [3, 4]. However, many members of the public only weakly attend to such messaging [5]. It is likely, therefore, that a substantial proportion of the public comes into contact with policy messaging indirectly through interpersonal interactions in which policy preferences are shared in conversation or through social media posts by peers. It is important for scholars to understand not only the effects of direct appeals by elites on those who consume this messaging, but also the indirect effects that may occur through subsequent interactions among the public.

A heightened understanding of the direct and indirect effects of elite messages on individuals’ policy attitudes has meaningful consequences for democratic governance. Political elites and policy stakeholders who seek to inform the public can be aided when their messages are shared between social contacts. On the other hand, false or misleading information from elites may be rapidly disseminated through social networks, both in-person and on-line, leading the public to become misinformed about policy issues. These effects may have downstream impacts on behavior, such as voting, and attitudes, such as affective polarization.

Here, we investigate both the direct and indirect impact of elite messages on individual public opinion. Direct appeals from political elites and policy stakeholders constitute the messages that they communicate themselves, whereas indirect appeals are those messages that are filtered through an individual’s social contacts. Our research design enables us to observe and analyze a complex messaging environment that, although carefully controlled in a lab, contains many of the features that messaging both from elites to the public and peer-to-peer interactions would have in a natural environment.

Drawing from a series of experiments on student organizations at a large Midwestern research university, we seed elite messages on space privatization across experimental rounds and track how those messages spread across real world social networks—of people who actually know each other and meet in person—in real time. We find strong evidence that impersonal messages from political elites and policy stakeholders do influence individual policy attitudes in a setting where individuals knowingly share their responses with friends in their social network. However, we find *no* evidence that elite messaging impacts policy attitudes when filtered through social contacts. Our results have implications for how we understand public opinion—as both exercising influence on politicians and as endogenous to those politicians themselves.

## Direct & indirect effects of elite manipulation of public opinion

The extent to which public policy is responsive to public opinion—“the global policy preference of the American electorate”—is hotly debated [6, p. 543]. Some argue that there is a high degree of congruence between public opinion and public policy [6]. Others are more skeptical, pointing toward weak opinion-policy links and toward elite manipulation of public opinion in the first place [7, 8]. Others operate under a contingent approach, which demonstrates a strong opinion-policy link under certain conditions with some issues and a weaker link under other conditions and other issues [9].

Although the magnitude of the effect of opinion on policy is debated, the effects that elites have on individual policy attitudes is better understood. Elected officials and other elites seek to influence the constituents’ attitudes, sometimes using “public opinion as a weapon of political struggle” [1, 2, 9, p. 657]. The “extensive efforts and frequent successes” of elites in shaping and manipulating public opinion are employed by presidents and other elected officials, governmental agencies and organizational bodies, and business elites and interest groups, among others [3, 9, p. 647].

Elites use various methods to influence Americans’ policy preferences. Some run their own public opinion polls to understand popular sentiment on certain issues. These polls often serve as tools with which politicians test the popularity of particular policy messages [2]. The role of president, especially, has broad institutional advantages with which he can increase the salience of a particular issue and even change public opinion itself [3]. Major governmental organizations have their own public relations arms to promote their priorities and activities [10]. Business elites and interest groups seek to influence public opinion via think tanks, policy formation, and corporate control over the media, among other methods [11].

However, whether these efforts to impact public opinion are successful is difficult to demonstrate empirically. First, measuring elite messages and determining their effects on individual policy attitudes is difficult in observational settings [12]. Second, because elites are often divided on the issues, individuals are exposed to different and competing elite messages, making it difficult to understand their effects [13]. Third, issues vary in their salience and content, which complicates our understanding of elite influence on opinion. Finally, messages from policy stakeholders are often not directly absorbed by individuals but are mediated by the media and individuals’ social environment [14].

Accordingly, the effectiveness of elite policy messages must be evaluated in light of how they are absorbed by and spread across the social networks in which individuals are embedded. Taken together, we expect elites to *directly* influence individuals’ attitudes about political issues and also to have an *indirect* effect on public opinion, whereby individuals not directly exposed to a policy message can be influenced by it through social contacts.

Social networks are important qualifiers of elite effects on public opinion because they promote learning through the sharing of information. That networks facilitate the spread of information is not constrained by context or domain, and can include information related to health behaviors [15, 16], misinformation [17], and political information [18–20], among many others [21].

While individuals can be randomly assigned to social networks in an experimental setting, these networks often do not exist in the real world (but see [22] and [23]), bringing into question the external validity of the results. Additionally, results from such online studies may not generalize to face-to-face interactions if individuals share information anonymously, facilitating different behaviors than when individuals communicate with people with whom they have relationships. Relationships that exist outside the experimental setting are important modifiers of intra-network attitudes because individuals come into interactions having preconceived ideas about the knowledge and beliefs of their social contacts [13]; this information cannot be replicated in anonymous online experiments among strangers.

## Design

This study was approved by Ohio State’s University Institutional Review Board (IRB), protocol 2016B0490: We received online consent from participants. We conducted an experiment to understand how direct and indirect elite messages influence individual policy positions for individuals in a social network. To achieve our goal, we need to identify natural networks, measure network members’ existing attitudes toward policy, and monitor relevant information communication between closest peers. Observational studies can typically neither adequately track information sharing nor monitor this process in real time in a social network. Our study’s randomized treatment with experimental control is a key strategy that can give us causal identification and leverage over these questions.

### Participants

Participants were part of student organizations that were registered at a large Midwestern university. All registered organizations (more than 1,300 in total) were recruited for screening via email. Only groups with self-reported membership between 15 and 30 (likely with strong interpersonal relationships) and who could guarantee synchronous study participation of at least 10 members between February and April of 2017 (for feasibility) were eligible to enroll. Compensation was two-tiered, with participating organizations receiving $100 for their organizational budget and students $10.

### Design & treatments

Each organization’s members participated in a distinct experimental session. At the start of each session, each participant was randomly assigned to a pro-government or pro-private investment in space treatment, which involved viewing deferentially slanted statements regarding space-related investment (described below). As opposed to group-based randomization schemes (such as ego-centric or cluster randomization), this individualized randomization gains efficiency by taking advantage of our highly structured system of information transfer to understand indirect treatment effects. This scheme also produces un-patterned assignments intended to mimic a real world setting with a diversity of information sources among friends, as is reasonable for low-salience topics.

While space and space policy have recently become more salient with the bipartisan creation of the United States Space Force in December 2019 and a successful private manned space mission in May 2020, at the time this study was fielded, space policy was not an area of significant public concern. The fact that space policy was of low salience at the time of study was a deliberate choice and is important because it means that (as confirmed in our analysis) most participants did not enter the study with preconceived notions or strong, entrenched attitudes on the subject, thus allowing us to tap the dynamics of influence and learning.

**Fig 1:**
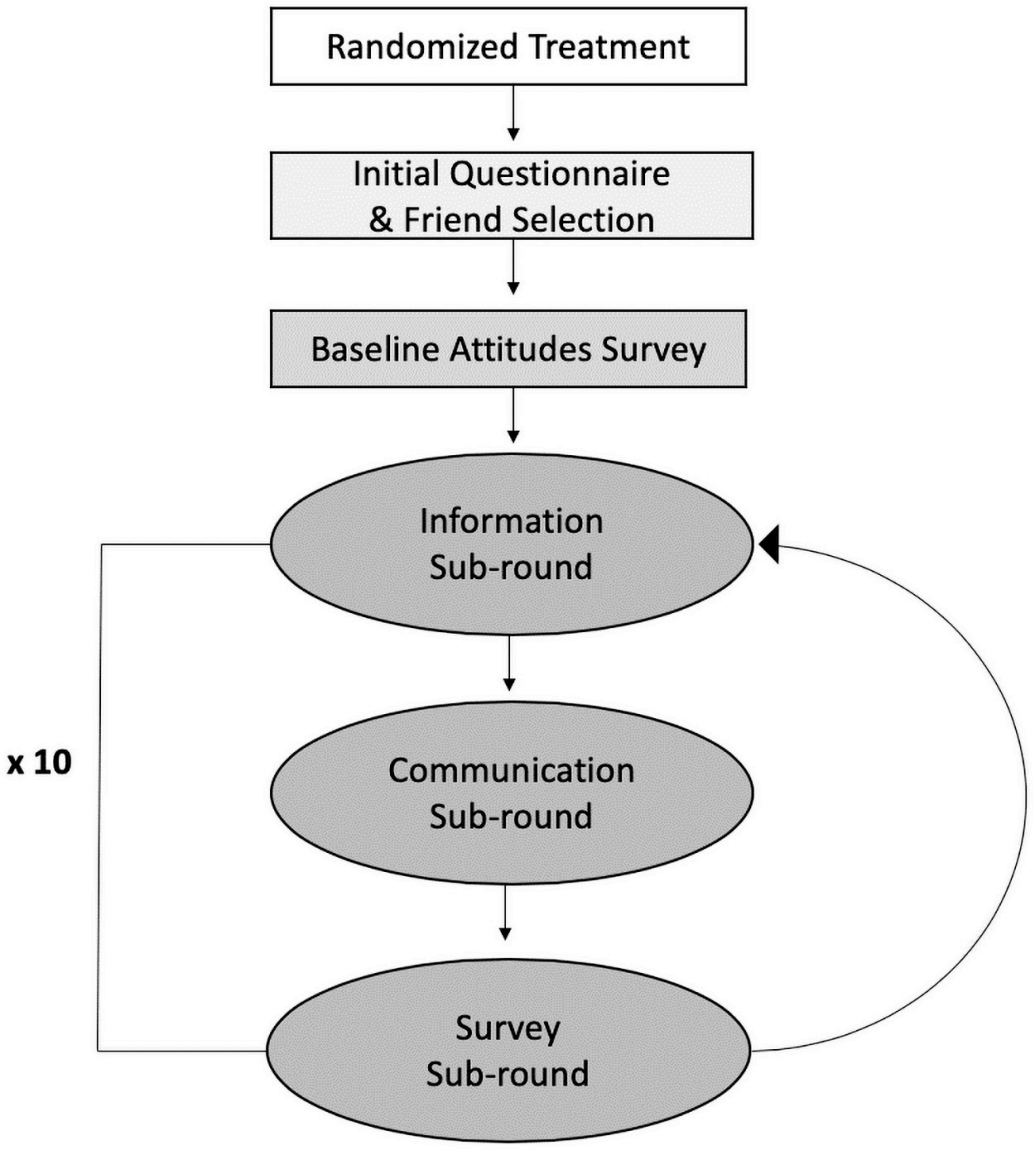
Diagram of individual experimental experience. Each round of the experiment consisted of three parts, including the information, communication, and survey sub-rounds.

Individuals completed the study in-person using tablets, connected via wifi and using the oTree software [24]. As depicted in Fig 1, participants were first asked to complete a standard set of demographic questions (see Appendix 1 Table 1 in S1 File) and provide a name by which other individuals in the group would recognize them (initial questionnaire and friend selection). Participants were then asked to select the names of exactly three “friends” in the group from a list of compiled names. These relationships are directional such that if Mark selected Megan as a friend, but Megan did not select Mark, Mark would see Megan’s information (as described below) but Megan would not see Mark’s information.

Next, participants were asked to respond to a battery of 11 questions (baseline attitudes survey), included in Appendix 3 in S1 File. Six of these questions were factual political questions; the other 5 were questions related to United States space policy. Participants’ answers to these questions provide a baseline of their political knowledge and space policy attitudes that we compare to responses to these same questions after they have been exposed to the experimental treatment. The 6 political knowledge questions were included in order to obscure the intended purpose of the experiment.

**Fig 2:**
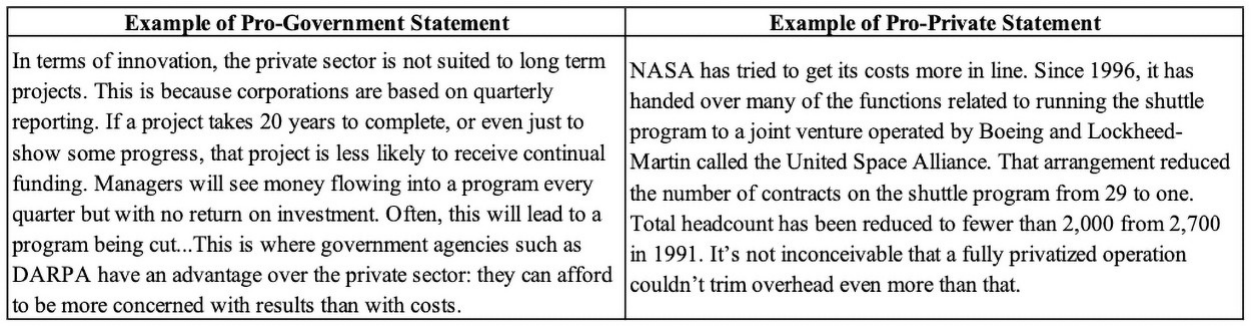
Examples of pro-government and pro-private investment in space statements utilized in experimental treatments. Individuals were randomly assigned to receive either four statements in support of government investment in space (left panel) or four statements in support of private investment in space (right panel).

After this baseline assessment, ten experimental rounds began. Each round consisted of three parts, or sub-rounds. In the first sub-round, called the “information sub-round,” each participant was shown one piece of information, randomly chosen (without duplication) according to their treatment assignment. Across the ten rounds, those assigned to pro-government treatment viewed arguments in support of increasing government involvement in space and six political facts. Those assigned to pro-private treatment viewed arguments in support of increasing space privatization and the same six political facts. See Fig 2 for examples of these and Appendix 2 in S1 File for all 14 statements.

The second sub-round, called the “communication sub-round,” gave participants the opportunity to view the responses to the prior survey sub-round given by the other participants selected as their three “friends”. This sub-round allowed for the social transmission of political beliefs. In the third sub-round, the “survey sub-round,” participants were asked to answer the same battery of 11 questions probing their political knowledge and opinions on privatization of space.

After this round, participants were again seeded new information from political elites (a new “information sub-round”), starting the next round of the experiment. The three sub-rounds (information, communication, and survey) comprise a full round of the experiment. For each session of the experiment we conducted 10 full rounds. At the end of the experiment, participants were provided with all of the correct factual information and debriefed.

### Pro-government & pro-private measure

The attitudes survey completed at baseline and in each “survey sub-round” included four questions that summarize respondents’ opinion of government investment in space exploration, both for and against, the wording of which is provided in Appendix 2 in S1 File. We summarized the strength of each participant’s pro-government opinion in a single composite score by averaging scores from these four questions (details are provided in Appendix 3 in S1 File). A participant that is strongly pro-government investment would have a composite score close to one; participants that are strongly pro-private investment would have a composite score close to -1. Participants with scores close to 0 do not strongly favor a single source of space funding. At each round, we compared each participant’s composite score to their composite score at baseline, and term this difference the ‘shift’ in score, which can range from -2 to 2. We summarize the scores and shifts across all the networks with a simple average among relevant subsets of participants so that large networks naturally ‘count’ more than small networks.

At baseline, the average space composite score was -0.04, indicating that on average, at the outset of the survey, participants do not favor investment from one source over the other (see Appendix 3 Fig 1 in S1 File for the full distribution). The distribution of this shift from baseline to round 10 is provided in Appendix 3 Fig 2 in S1 File. It depicts a distribution in which not very many participants changed their attitudes about government involvement in space (shift is close to zero), some participants changed their attitudes a little, and a few participants changed their attitudes substantially. Accordingly, participants may not be very swayed by official or social information—perhaps because they are not absorbing or do not value this information or because attitudes about space privatization are more entrenched than originally expected.

Of course, there are other possibilities that may explain why relatively few participants changed their attitudes about space privatization in addition to the two previously discussed. It is possible that the space treatment statements themselves were weak or ineffective, that participants did not find the official sources of information about government involvement in space to be credible or to be sharing valuable information, or that asking participants to commit to attitudes about space up front caused them to entrench their attitudes to remain consistent throughout the experiment. All of these possibilities are consistent with our data.

### Statistical analysis

Our analysis tests for the effect of treatment assignment conditional on the participant-generated networks. Because of our pre-defined limits to the network topology, our study is in effect a 2x4 factorial experiment, where the first factor (elite information) is the individual’s assigned set of elite arguments and the second factor (social information) is the number of chosen friends who were assigned to pro-government treatment (0 to 3). Unlike a non-network experiment, connections between individuals in the present study are likely to induce dependencies among responses that would be difficult to capture in a parametric model. Instead of attempting such modeling, we gauge the statistical significance of observed treatment effects (versus the null hypothesis that a treatment has no effect) conditional on the observed network topology by permuting individual treatment assignments within networks [25]. These permuted assignments then ‘trickled down’ to alter the assignments of the chosen friends so that each permutation simultaneously changed both experimental factors. See Appendix 11 in S1 File for more details about the permutation-based statistical procedures. In all the following analyses, we used the same 5000 permutations to construct relevant reference distributions. We report estimated p-values relative to the permutation reference distribution and observed functional averages relative to functional boxplots.

## Results

Of the 460 organizations whose leadership completed the screening survey, 30 ultimately scheduled sessions and participated in the study between February and April of 2017. Data from three organizations were unusable due to difficulties with technology at the time of data collection (1 organization) and baseline participant engagement (2 organizations). The remaining 27 groups constituted 358 participants and represent a diverse array of interests including academics, agriculture, and cultural and performing arts. After accounting for missing data, 248 individuals contributed to statistical analyses. We found no evidence that individuals omitted from analyses had been assigned treatments unevenly across levels. Permuted datasets were subset for complete cases after all participants’ assignments were permuted. Appendix 5 in S1 File details recruitment and participation rates and tests for missing data patterns.

**Fig 3:**
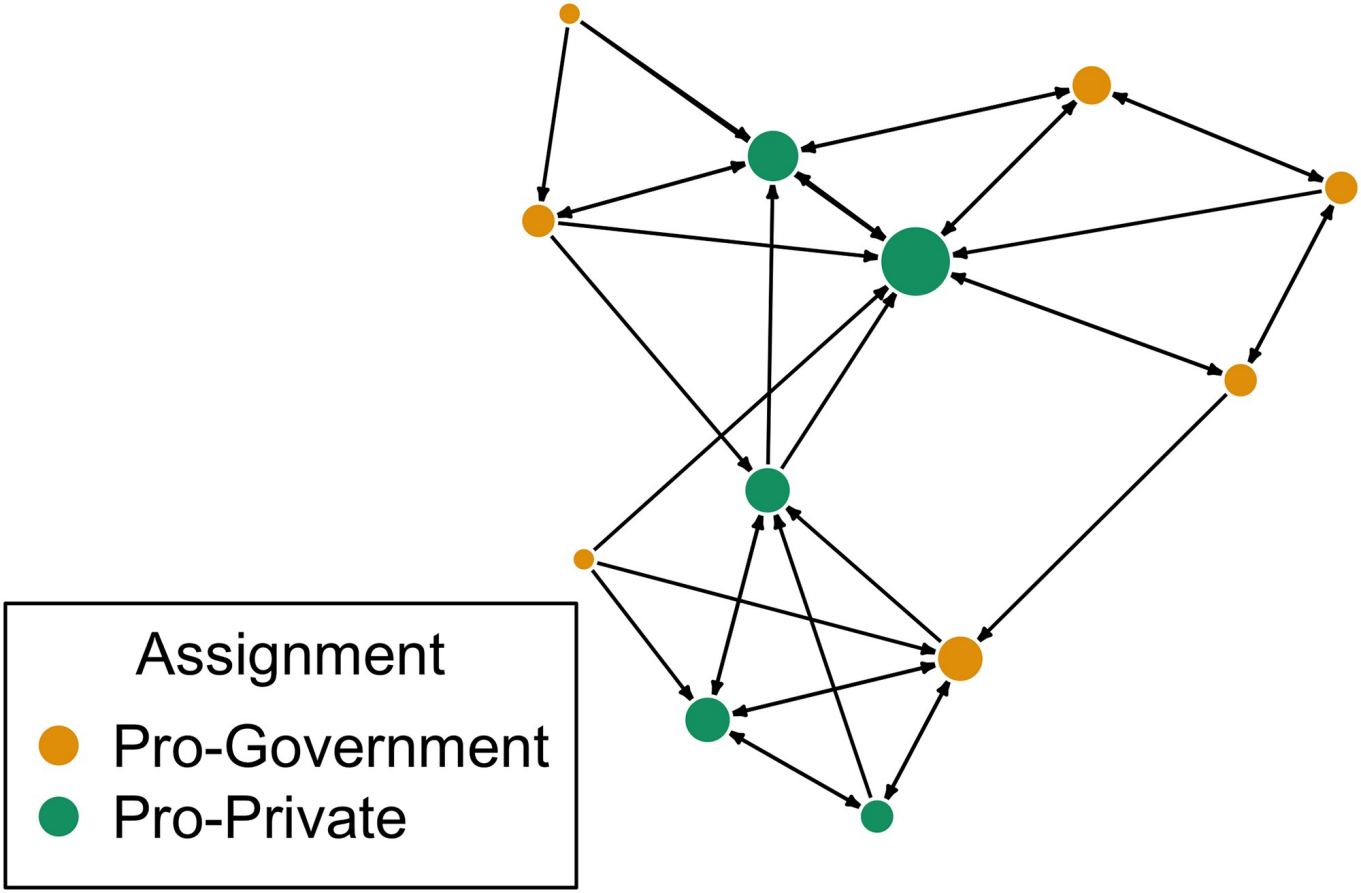
An example of one of the student organizations in our study.

Fig 3 provides an example of one of these networks that includes 12 participants. Each circle represents a participant, or node, which is colored by treatment assignment. The connections between participants are directed: each participant selects three friends in the group and is simultaneously chosen by other members of the group. The bigger circles correspond to those participants who were selected by their group members more often than others.

It is possible that individuals select friends with similar attitudes on space privatization. Although we do not expect homophily in attitudes about space privatization due to the low salience of the issue in our student sample, we want to ensure that any social (indirect) effect that we find is a result of influence. In Fig 1 of Appendix 6 in S1 File, we show the association between participants’ baseline space composite score and their friends’ baseline composite scores. We see little evidence for any homophily based on space investment preferences at baseline.

Table 1 shows the cross-classified counts of participants assigned to each treatment versus their friends’ assignments for all study participants for which we have complete data. A permutation-based p-value of 0.80 confirms no evidence of unintentional imbalance in treatment assignments.

**Table 1:** Number of participants with complete longitudinal data (including friend data) in each cross-classification defined by the official information assignment and their friends’ assignments.

Official Information	Pro-Government Friends				Total
	0	1	2	3	
Pro-Private	21	52	46	9	128
Pro-Government	19	57	37	7	120

### Marginal effect of elite information

In this section, we consider how patterns of participant composite scores differ by their assignment to view pro-government or pro-private investment official information. That is, we only consider the participant’s own assignment, and not the cascading effects of assignment filtered through social interactions. Because treatment assignment is random, we expect the indirect influence of treatments assigned to friends to average out across groups defined by an individual participant’s treatment assignment. See Appendix 11 in S1 File for further discussion of the interpretation of marginal effects.

For the direct messages individuals receive from political elites (official information marginal effects), we expect that participants who view the pro-government elite information will tend to have composite scores that increase (relative to baseline) across the rounds. That is, on average, score shifts are expected to increase across rounds. We expect an opposite effect for those in the pro-private official information treatment; score shifts are expected to decrease across rounds. we demonstrate that the treatment assignment is “random” in the sense that pre-poster viewing composite scores do not systematically differ by treatment group.

**Fig 4:**
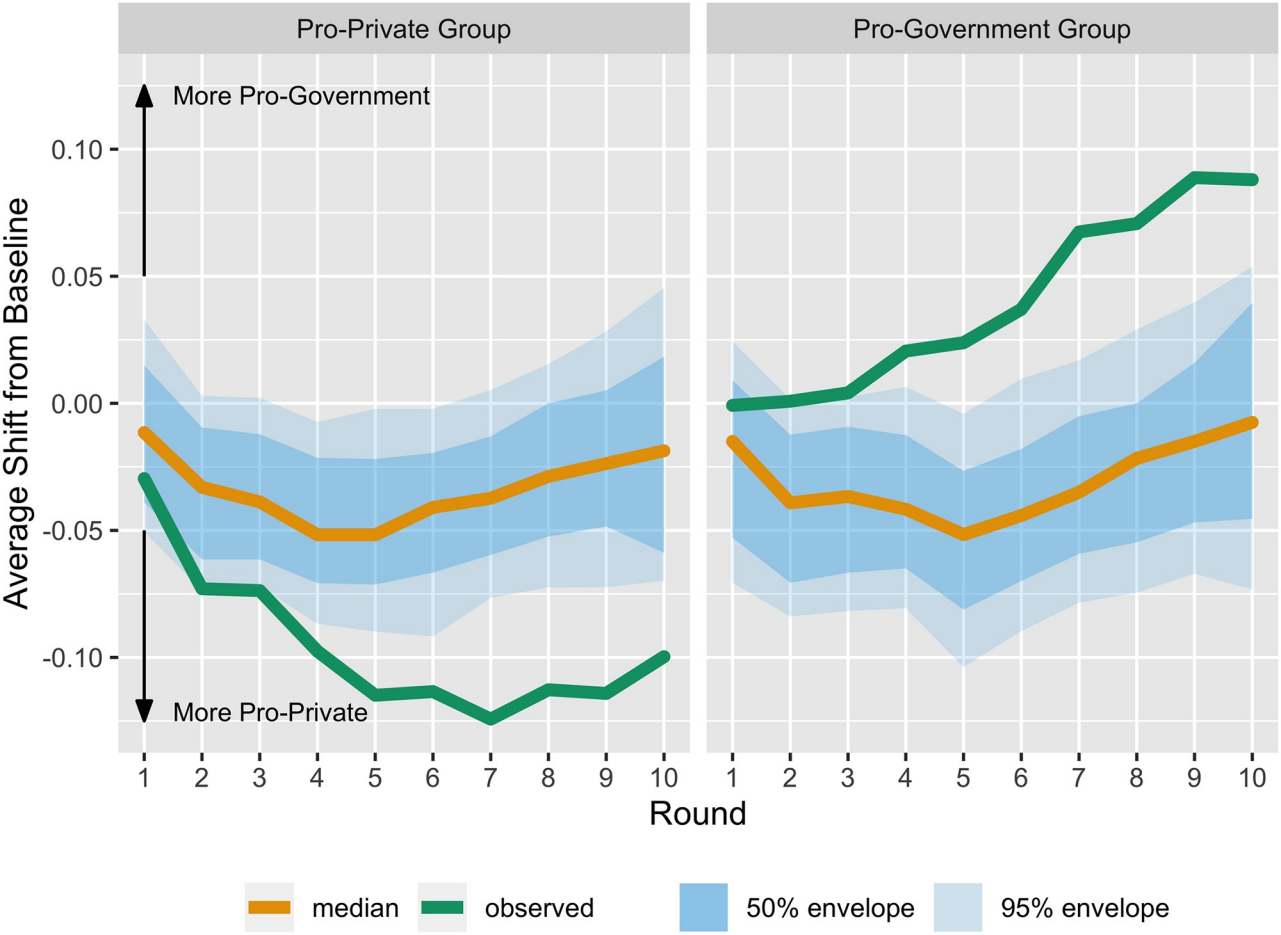
Marginal score shift from baseline by assigned official information treatment group. The green line in the left panel displays the progression across the 10 rounds of the average score shift from baseline (time 0), for those assigned to view proprivate funding messages, disregarding the possible indirect effect of messages shown to friends. Negative score shifts indicate that on average participants in this group express opinions that are more proprivate than they had at baseline. The orange lines are median (most usual) progressions and blue bands are 50 and 95 percentile envelopes constructed via the permutation reference distributions for these same progressions. The right panel displays the same attributes for the group randomly assigned to view progovernment messages.

We next examine the longitudinal average score shifts across the rounds, separated by and compared across the official treatment assigned to each participant. Fig 4 shows the average shift from baseline for the pro-private investment and the pro-government investment treatment groups (left and right panels, respectively). The green line in each depicts the observed progressions of this average across rounds. The orange lines (median progressions) and blue bands (percentile envelopes) correspond to the permutation reference distributions for these same progressions. They are constructed as follows. For each permuted dataset, the average shift progression across rounds is calculated to create a collection of progressions. The “usualness” of each progression in this collection is calculated based on a modified band depth method [26, 27]. The median curve (orange line) is the most usual curve in the collection of progressions. A 50% envelope is the narrowest band that fully contains the most usual 50% of the curves. A 95% envelope correspondingly contains all but the 5% most unusual progressions.

Thus, we see from the left-hand panel of Fig 4 that the observed trend in average shift from baseline toward more pro-private investment opinions (negative scores) is more unusual than at least 50% of permuted pro-private treatment group progressions after the first round, and more unusual that at least 95% of the progressions after the third round. The right-hand panel of Fig 4 shows a similarly unusual observed trend in average shift from baseline toward more pro-government investment opinions (positive scores). In short, starting in round four, shifts from baseline are more negative (positive) for those receiving pro-private (pro-government) investment official information than would be expected by chance. Because the official information was presented in random order, the number of pro-government or pro-private statements that we expect participants to see by round four is 1.6 items. We expect only 1 in 14 to have not viewed any such statements within the first four rounds.

Because the observed average score shifts from baseline are outside of the distribution that we would expect by chance, we can reject the null hypothesis and conclude that the pro-government (pro-private) investment elite treatment does increase (decrease) average composite scores across experimental rounds. Elite policy messages do impact individual policy attitudes. We are confident in this conclusion due to our ability to directly measure elite policy messages, the randomization of those messages in an experimental setting, and that we find results despite the low number of observations. A statistically significant difference between the two treatments (i.e., a marginal effect of elite information) is confirmed in Appendix 7 Fig 2 in S1 File.

### Effect of social information

As previously argued, we also expect elite policy messages to operate indirectly, through individuals’ social networks. We have already seen that official information affects participants’ opinions, on average. Thus, we expect that participants with more friends (out of three) who were assigned to view pro-government investment information may adopt these friends’ presumably more pro-government investment opinions.

**Fig 5:**
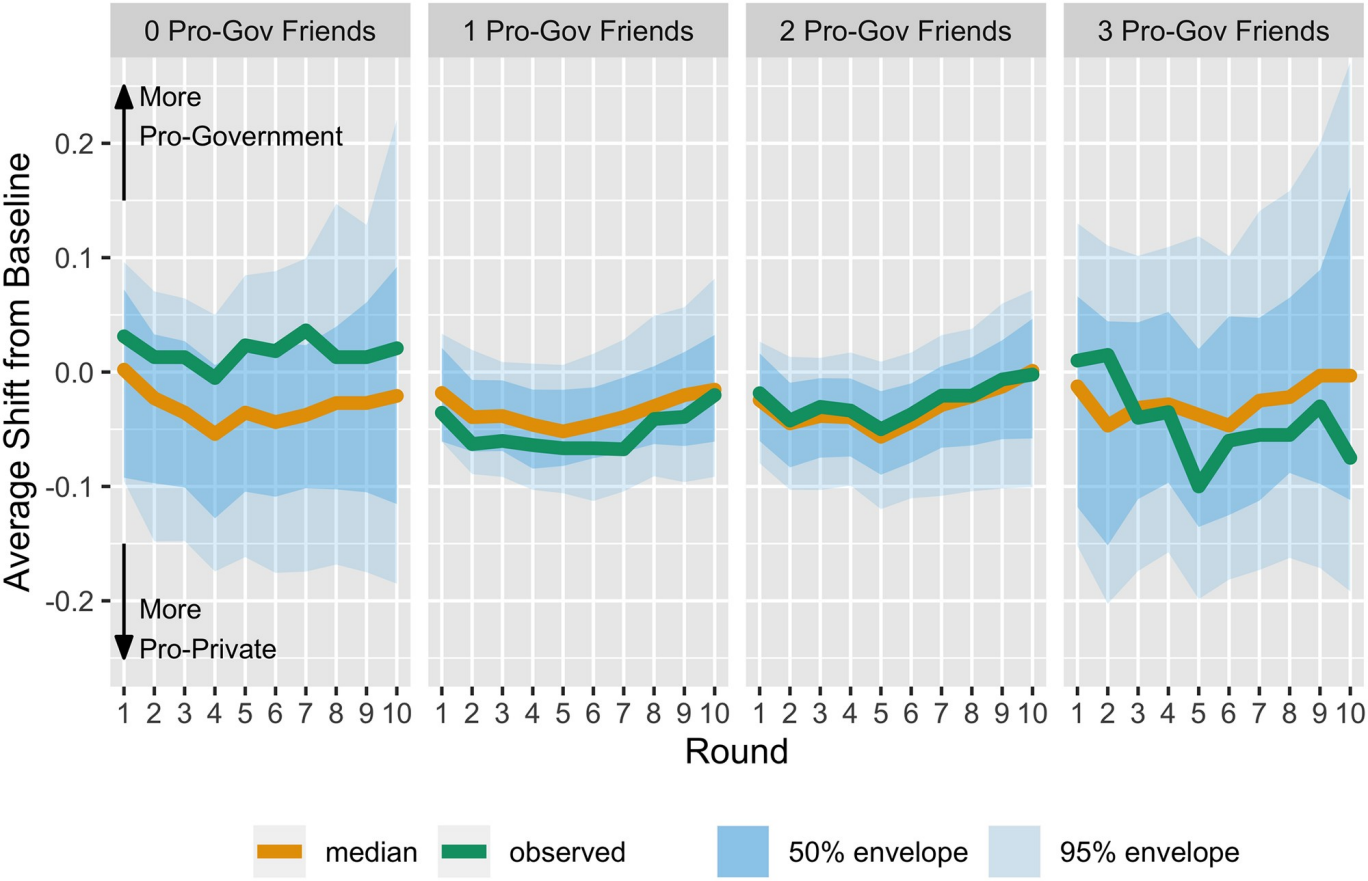
Average shift from baseline by social information treatment group. The green line in the left panel displays the progression across the 10 rounds of the average score shift from baseline (time 0), for those with none of their three selected friends assigned to view pro-government funding messages, disregarding the possible direct effect of messages shown to that individual. Negative score shifts indicate that on average participants in this group express opinions that are more pro-private than they had at baseline. The orange lines are median (most usual) progressions and blue bands are 50 and 95 percentile envelopes constructed via the permutation reference distributions for these same progressions. The other panels display the same attributes for the groups with 1, 2, or 3 of their selected friends assigned to view pro-government messages.

Fig 5 displays the progression of average score shifts from baseline among groups defined by the number of friends (out of three) that have been assigned to view pro-government investment information. For each group, we see that the observed average shift progression (green line) falls entirely or nearly entirely within the 50% envelope that describes the most usual half of the permuted progressions. Though not nearly statistically significant, the zero pro-government friends and the one pro-government friend groups may have shifts from baseline that trend high or low due to their baseline values trending low and high, respectively (see Appendix 8 Fig 1 in S1 File).

Accordingly, on average, pro-government (pro-private) messages seen by individuals’ friends do not significantly change their own attitudes in the pro-government (pro-private) direction more than would be expected by chance. These results may suggest a lack of indirect effects of elite messages on individual policy opinions or may indicate a lack of power to detect the effect of socially shared opinions as filtered through friends’ official information assignments. Low power may have been exacerbated by the small number of individuals assigned to treatments where we would expect the largest marginal effect—having zero or three pro-government friends. We undertake two further analyses to explore the possibility of trends related to social information that may be revealed by secondary analyses.

First, learning in social networks could be facilitated by *specific* social contacts and not all of them—what we term the most “popular”, or influential, friends. Focusing on these individuals may reveal effects of social information masked by combining such popular individuals with the rest of the friends. In short, we do not find strong evidence of the effect of a popular friends’ assignment on individuals’ own policy attitudes (see Appendix 10 in S1 File).

Second, it could also be the case that the effect of receiving the direct pro-government informational treatment is magnified by also having more friends assigned to view pro-government elite policy messages. In other words, we expect that the average size of the difference in score shifts for those assigned to view pro-government information versus pro-private information will vary according to the number of chosen friends assigned to view pro-government information. The symmetry of our random treatment assignments might result in the presence of such an interaction effect even without a marginal effect of social information.

**Fig 6:**
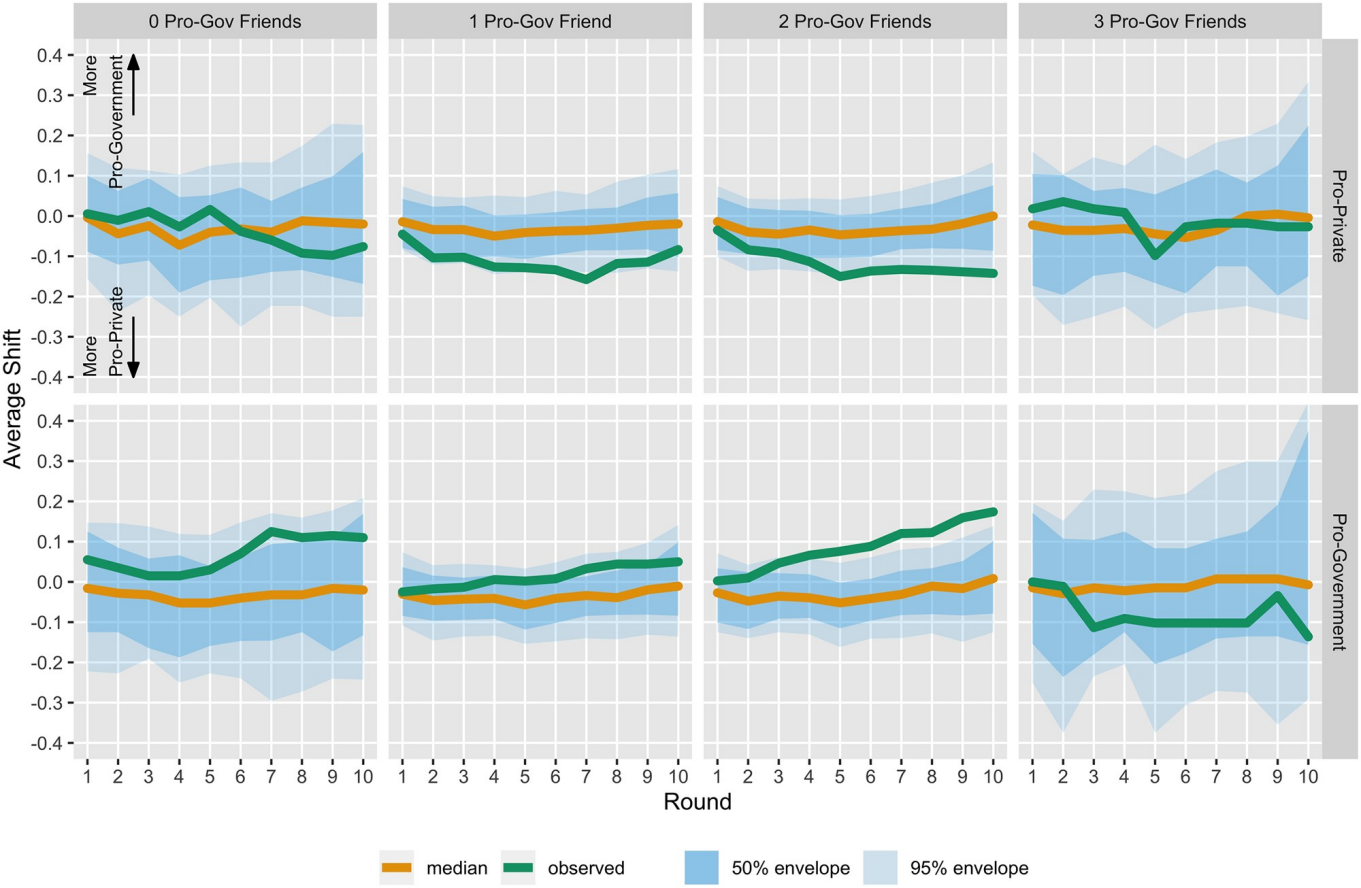
Average shift from baseline by official and social information treatment groups. The green line in the upper left panel displays the progression across the 10 rounds of the average score shift from baseline (time 0), for those both assigned to view pro-private messages and for whom none of their three selected friends assigned to view pro-government funding messages. Negative score shifts indicate that on average participants in this group express opinions that are more pro-private than they had at baseline. The orange lines are median (most usual) progressions and blue bands are 50 and 95 percentile envelopes constructed via the permutation reference distributions for these same progressions. The other panels display the same attributes for the other five strata defined by the cross-classification of direct and friend treatment assignments.

Fig 6 displays the progression of average shifts across rounds within cross-classified groups. For most of these smaller groups, the shifts are not distinct from the progressions after permuting assignments. The one exception happens to be for those assigned to view pro-government investment messages and for whom two of their friends are also assigned to view pro-government investment messages. But, with so many comparisons this single statistically interesting progression may simply be a chance outlier that is not supported by broader trends across the groups. Further, note that the envelope widths for some of these small groups are at least double those observed in the marginal cases, demonstrating the sharp loss of power for these more detailed analyses (as depicted in Table 1). Accordingly, we do not see strong evidence for an interaction effect whereby elite policy messages are magnified by pro-government social information to increase shifts over time (see also Appendix 9 in S1 File).

Although the results displayed visually in Fig 6 are not statistically significant, we note that the pattern of results may be consistent with the effects of official information overcoming any effects of social information. Indeed, the middle panels (those with social contacts whose official information are disagreeing), which are those with the highest number of participants due to the nature of the experimental design, show that participants’ attitudes are generally in line with the official information presented by the participant’s own condition regardless of the conditions of their social contacts. The outside panels show much less evidence of this, but that may be a reflection of the lack of statistical power in these comparisons. Finally, we do not see even weak evidence of a “dose response,” in which the number of friends whose condition is the same as the focal participant’s bolster the effect of the official information. Importantly, none of these relationships are statistically significantly different from baseline, and although there is some suggestion of a difference among the portions of our sample with the most power these relationships are not distinguishable from chance.

Because none of our analyses of various possible ways social information may affect individual policy opinions yielded strong evidence of such an effect, we are more confident in our null results even in the face of low power. Future work should address the many channels in which friends may influence individual policy opinions and should consider our results in designing future studies to provide ample power to detect small effect sizes.

## Discussion

Utilizing novel experimental data of real-world social networks, we demonstrate the power of appeals from political elites and policy stakeholders in influencing individual policy attitudes in a setting where these attitudes are shared within the peer network. However, we were unable to find evidence of indirect influence of elite policy messages, via social networks. That we did not find evidence of indirect social effects was surprising to us. Even when we analyzed information shared by the most influential (or popular) friends, we found no evidence that elite messages filtered through an individuals’ social network impact policy attitudes. This may either be because of the small sample size (low power to find effects), because indirect effects on policy attitudes are quite small, or perhaps the nature of the interaction in our experimental setting is different enough from real-world interactions to depress any social effects. Either way, we expect that the indirect effects of elite messages in the real world to be minimal on average, especially when messages from friends are divided—which our experiment simulated. In cases where social messages are more consistent, it is possible this would produce larger effects. Future work should explore the alternate channels in which social networks influence individual policy attitudes.

As mentioned previously, we deliberately chose to investigate participants’ attitudes about the privatization of space exploration. Space privatization represents a nascent political issue because it is of low salience and is not particularly polarized across the political spectrum. Specifically, a 2015 Pew Research Report found little partisan difference on whether or not the federal government should be involved in space exploration [28]. Accordingly, we do not necessarily expect the findings to translate to issues where individuals hold more entrenched attitudes such as gun control and abortion rights. The decision to use space privatization may mean that participants’ attitudes may be more likely to change throughout the course of the experiment. However, we do not expect this change to be more or less pronounced in response to official over social information.

This work addresses some of the difficulties around whether elite attempts to influence public opinion are successful. First, we address measurement difficulties by using a controlled, experimental setting. Second, we seed competing elite policy messages (pro-government investment vs. pro-private investment in space) to mimic the divisions among elites as well as competition for influence. Finally, by focusing on a non-salient, non-controversial issue, our findings can generalize to a plethora of similar issues that elites seek to frame.

The normative implications of elite influence on public opinion assume even greater urgency in light of these results. On the one hand, elite policy messages serve to educate the public by providing correct and useful information about policy. On the other hand, information received from elites can be false, misleading, and/or biased. If elite policy messages are effective in changing individual policy attitudes, which our findings demonstrate, then efforts to educate the public can help individuals make more informed policy decisions, but efforts—intentional or otherwise—to mislead or manipulate the public can lead individuals to make less informed or “incorrect” decisions. Due to the powerful influence of elite policy messages on public opinion, we would agree with solutions that hold elites more accountable for the information they share.

## Supporting information

S1 File Appendices 1–12. (PDF)
