# Supplementary material for: Exploring the direct and indirect effects of elite influence on public opinion
Source: PLoS One. 2021 Nov 19;16(11):e0257335. doi: 10.1371/journal.pone.0257335 (PMC8604325; doi:10.1371/journal.pone.0257335)
Supplement: S1 File — (PDF) [file pone.0257335.s001.pdf]

Supplementary Appendices

Appendix 1. Participant Selection & Demographics

Leaders of the universe of student organizations at a large Midwestern university were contacted and invited to participate in a short survey. These representatives, most often group presidents, were asked to complete a six-question survey to determine the eligibility of their group to participate in the study. The survey asked about the size of their group as well as their willingness to devote group meeting time to the study. We originally contacted the presidents of more than 1,300 student groups. Of those presidents, 460 responded to the initial survey.

Groups with self-reported memberships of between 15 and 30 students who indicated initial willingness to participate in a research study were contacted via e-mail and a group meeting organized. In total, the research team met with 30 student organization groups between February and April of 2017. Three networks were not viable. One groups was not viable due to technology failure, and the remaining two were not viable due to participants not properly selecting alters. Groups that participated in the experiment received a $100 payment to their school organizational budget – conditional only on them getting ten group members to attend the meeting. In addition, each individual who participated was compensated with $10 to their university student account.

Among the viable networks, there were 358 participants, 33 of whom had some missing values in their longitudinal responses and 110 of whom had some missing values in their own or their chosen alters' responses. Appendix materials display the number of participants for all 27 networks when accounting for all the data (n=358), all ego complete data (n=325), and all ego and alter complete data (n=248). Participants with any missing longitudinal values were not included in the test statistics described in text, but were included in the creation of the permutations so that network structure was preserved across all permutations.

Networks ranged in size from 9 to 26 participants. The distribution of network size for the unadjusted networks and for the subset of each network where each participant contributed complete

answers in all 10 rounds is shown in Figures Appendix 1 Figure 1 and Appendix 1 Figure 2. Participant demographics are available in Table 1 below.

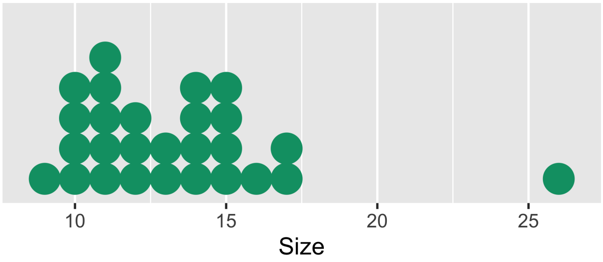

Appendix 1 Figure 1. Network sizes.

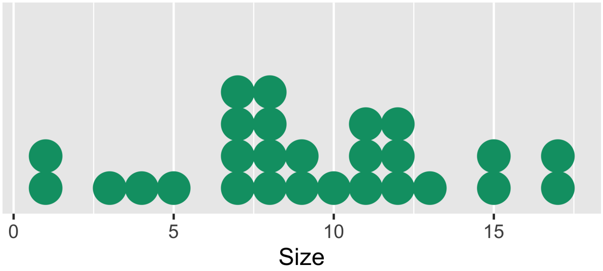

Appendix 1 Figure 2. Network sizes, after removing individuals with missing data.

Variable	Overall	Pro-Private	Pro-Gov
N	248	128	120
Race			
~~Asian Pacific Islander	41 (16.7)	21 (16.5)	20 (16.9)
~~Black/African American	24 (9.8)	14 (11.0)	10 (8.5)
~~Hispanic or Latino	9 (3.7)	5 (3.9)	4 (3.4)
~~Other	15 (6.1)	9 (7.1)	6 (5.1)
~~White	156 (63.7)	78 (61.4)	78 (66.1)
Gender			
~~Female	140 (56.9)	72 (56.7)	68 (57.1)
~~Male	102 (41.5)	53 (41.7)	49 (41.2)
~~Prefer not to answer	2 (0.8)	1 (0.8)	1 (0.8)
~~Prefer to self describe	2 (0.8)	1 (0.8)	1 (0.8)
Age (mean, SD)	20.73 (2.07)	20.63 (2.03)	20.83 (2.12)

Education			
~~High school graduate or GED includes technical vocational training that doesnt count towards college credit	97 (39.4)	52 (40.9)	45 (37.8)
~~Some college some community college associates degree	109 (44.3)	53 (41.7)	56 (47.1)
~~Four year college degree bachelors degree	29 (11.8)	17 (13.4)	12 (10.1)
~~Some postgraduate or professional schooling no postgraduate degree	9 (3.7)	3 (2.4)	6 (5.0)
~~Postgraduate or professional degree including masters doctorate medical or law degree	2 (0.8)	2 (1.6)	0 (0.0)
Party_ID			
~~Democrat	118 (48.6)	60 (48.0)	58 (49.2)
~~Independent	69 (28.4)	38 (30.4)	31 (26.3)
~~Republican	34 (14.0)	19 (15.2)	15 (12.7)
~~Something else	22 (9.1)	8 (6.4)	14 (11.9)
Ideology			
~~Conservative	20 (8.2)	12 (9.4)	8 (6.8)
~~Liberal	116 (47.3)	58 (45.7)	58 (49.2)
~~Moderate	74 (30.2)	38 (29.9)	36 (30.5)
~~Very conservative	7 (2.9)	3 (2.4)	4 (3.4)
~~Very liberal	28 (11.4)	16 (12.6)	12 (10.2)

Appendix 1 Table 1. Demographic Summary of participants with complete data, grouped by treatment

Appendix 2. Treatment Statements

Political Knowledge Statements:

1. Rex Tillerson is The Secretary of State.
2. Clarence Thomas is a Supreme Court Justice.
3. Mike Pence is the Vice President of the United States.
4. The Republican Party holds a majority in the U.S. Senate.
5. The Republican Party holds a majority in the U.S. House of Representatives.
6. The United States recently announced that it would re-establish diplomatic relations with Cuba.

Space Policy Statements:

Leading Question: Regarding The U.S. Commercial Space Launch Competitiveness Act, which encourages private sector investment in space exploration and streamlines commercial space launch activities.

1. **Pro-Government Statements**
   (a) Opponents of the bill say: “In terms of innovation, the private sector is not suited to long term projects. This is because corporations are based on quarterly reporting. If a project takes 20 years to complete, or even just to show some progress, that project is less likely to receive continual funding. Managers will see money flowing into a program every quarter but with no return on investment. Often, this will lead to a program being cut...This is where government agencies such as DARPA have an advantage over the private sector: they can afford to be more concerned with results than with costs.”

   (b) Monica Grady, professor of planetary and space sciences at Open University, Milton Keynes, UK says: “I am also concerned about statements ... made regarding public–private partnerships, and increasing the role of the private sector in space exploration... without sufficient thought for safety and governance...In my view, the international collaborative efforts that are currently in place are the correct way to progress.”

(c) Political columnist Charles Krauthammer says: “Sure, decades from now there will be a robust private space-travel industry. But that is a long time. In the interim, space will be owned by Russia and then China.”

(d) John Logsdon, former Director of the Space Policy Institute at The George Washington University says: “the principal benefits from human spaceflight are intangible, but nevertheless substantial.” The moon missions of the ’60s instilled in Americans a sense of “international prestige and national pride,” something Logsdon thinks is best produced by initiatives at the federal level.

2. **Pro-Private Statements**

(a) Supporters of the bill say: “This bill will unite law with innovation, allowing the next generation of pioneers to experiment, learn and succeed without being constrained by premature regulatory action...Virtually every space stakeholder group supports this bill. This bill encourages the private sector to launch rockets, take risks and shoot for the stars.”

(b) Avatar director James Cameron says: “The president and NASA have crafted a bold plan that truly makes possible this nation’s dreams for space. Their plan calls for the full embrace of commercial solutions for transporting astronauts to low Earth orbit after the space shuttle is retired this year. This frees NASA to do what it does best: deep space exploration, both robotic and human.”

(c) Foreign Policy’s Esther Dyson says: “The U.S. Defense Department may have created the Internet, but had it kept control of the technology, it’s unlikely the Web would have become the vibrant public resource it is today. That credit goes to the investment and activity of private citizens and private companies, starting in the late 1980s and early 1990s.”

(d) Supporters of the bill say: NASA has tried to get its costs more in line. Since 1996, it has handed over many of the functions related to running the shuttle program to a joint venture operated by Boeing and Lockheed-Martin called the United Space Alliance. That arrangement reduced the number of contracts on the shuttle program from 29 to one. Total headcount has been reduced to fewer than 2,000 from 2,700 in 1991. It’s not inconceivable that a fully privatized operation couldn’t trim overhead even more than that.

Appendix 3. Baseline Attitudes Survey & Composite Scores

These are the four questions utilized to establish participants' attitudes in support of private or government investment in space. [1] These questions were also asked in each survey sub-round.

1. Do you favor or oppose the U.S. Commercial Space Launch Competitiveness Act, which encourages private investment in space? (Pro-private investment)
2. Would you favor the U.S. government allocating billions of dollars to send astronauts to places like the moon, Mars, and asteroids? (Pro-government investment)
3. Do you think the U.S. military and intelligence agencies should setup plans for space exploration, the maintenance of strategic assets in space, and military engagement in space? (Pro-government investment)
4. Do you think the U.S. government should step back and rely more on private, rather than public, entities to run the country's manned space missions in the future? (Pro-private investment)

We summarized the strength of each participant's Pro-Government opinion in a single composite score by averaging scores from these four questions. As responses to the first question were on a 5-option scale, we computed a re-scaled score that ranged from -1 (strongly opposed) to 1 (strongly in favor).[2] The other three questions included 'Yes', 'No', and 'Unsure' response options. For the second pro-private question (Question 4), individuals who answered 'Yes' were given a value of '-1', 'Unsure' a '0', and 'No' a '1'. This scale was reversed for the second and third (pro-government investment) questions. Accordingly, a participant that is strongly pro-government investment would have a composite score close to one, and their counterparts that are strongly pro-private investment would have a composite score close to -1. At each round, we compared each participant's composite score to their composite score at baseline, and term this difference the 'shift' in score, which can range from -2 to 2.

[1] There was also a fifth opinion question that was not specifically pro-government or pro-private investment. We do not use that question for any analyses presented in this paper.

[2] Specifically, the scale included Strongly favor (-1), Favor (-0.5), Neither favor nor oppose (0), Oppose (0.5), and Strongly oppose (1).

Figure Appendix 3 Figure 1 shows the distribution of the composite scores at baseline, and Figure Appendix 3 Figure 2 depicts the shift in composite scores from baseline to round 10.

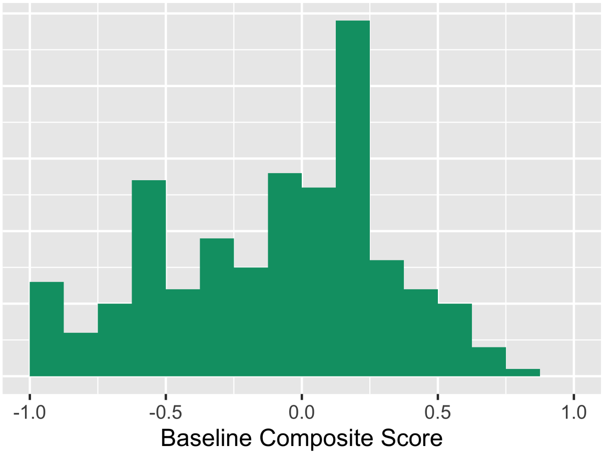

Appendix 3 Figure 1. Distribution of Composite Scores at Baseline.

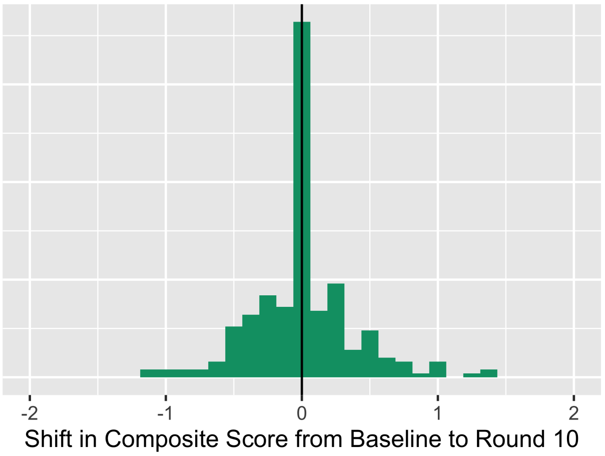

Appendix 3 Figure 2. Distribution of Shifts from Baseline to Round 10.

Appendix 4. Baseline Political Knowledge Questions

We asked participants the following questions to establish their baseline political knowledge. We have included the answer options in parentheses where the correct answer option is bolded.

> "We would now like to ask you a few factual political questions, along with your opinions on several issues relevant to U.S. space policy. Your answers to these questions will be shared with other participants in the study, and you will see other participants' responses as well. You will be presented with several pieces of media information regarding these questions as you go through these pages. Note that you might not see the same information as other people, therefore, some people might have information that you do not have. Also, the people that you share your answers with might not be the same people that are sharing their answers with you. Please click the next button to continue."

1. What office or political position does Rex Tillerson currently hold? (Secretary of Defense; Head of the DNC; **Secretary of State**; None; Don't know)
2. What office or political position does Clarence Thomas currently hold? (President; **Supreme Court Justice**; Speaker of the House of Representatives; Senator; Don't know)
3. What office or political position does Mike Pence currently hold? (President; **Vice President**; Senator; Member of Congress; Don't know)
4. Which political party currently holds a majority in the U.S. Senate? (**Republican**; Democratic; Neither holds a majority; Don't know)
5. Which political party currently holds a majority in the U.S. House of Representatives? (**Republican**; Democratic; Neither holds a majority; Don't know)
6. The United States recently announced that it would re-establish diplomatic relations with with of the following countries? (**Cuba**; Russia; North Korea; Yemen)

Appendix 5. Detailed Recruitment & Participation Rates

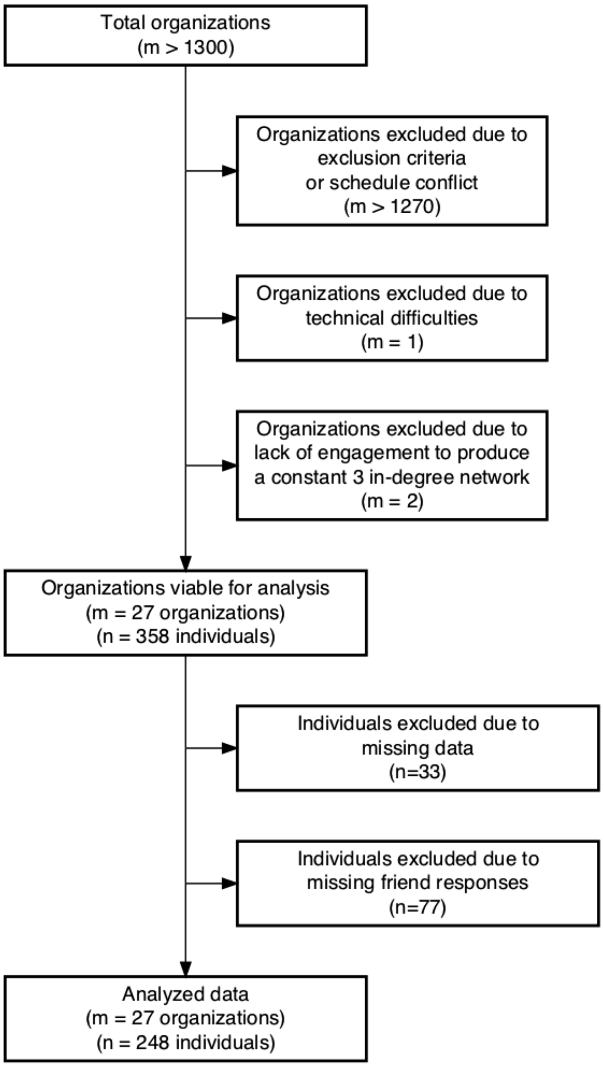

Appendix 5 Figure 1. CONSORT diagram.

Type of Missingness	Type of Treatment	CMH Test P-value
Participant Missing	Participant Assignment	0.83
Participant or Participant Friend Missing	Participant Assignment	0.30
Participant Missing	Number of Friends Assigned to Pro-Government Treatment	0.56
Participant or Participant Friend Missing	Number of Friends Assigned to Pro-Government Treatment	0.79

Appendix 5 Table 1. P-values from Cochran-Mantel-Haenszel tests of independence between missing data and treatment assignment, stratified by student organization. ”Participant Missing” signifies that the participant themselves are missing some data; ”Participant or Participant Friend Missing” signifies that the participant is not included in the analysis due to their own or their friend’s missing data. ”Participant Assignment” signifies the individual’s direct treatment assignment (pro-government or pro-private).

Appendix 6. Association Between Participant and Friends' Composite Scores

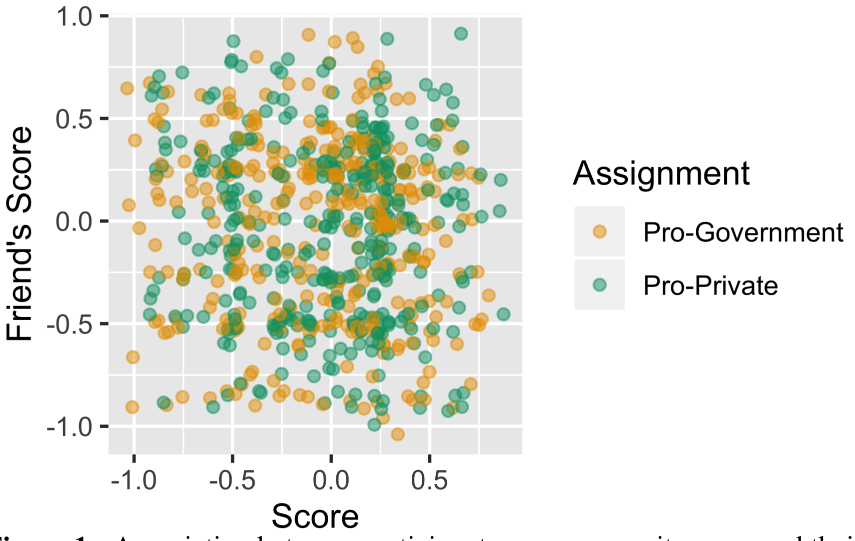

Appendix 6 Figure 1. Association between participant space composite score and their friends' composite scores. Includes individuals with complete and complete friend data. Plotted points are jittered to reduce obfuscation via overlap. Note that each participant is represented at least three times in each panel.

Appendix 7. The Marginal Effect of Elite Information

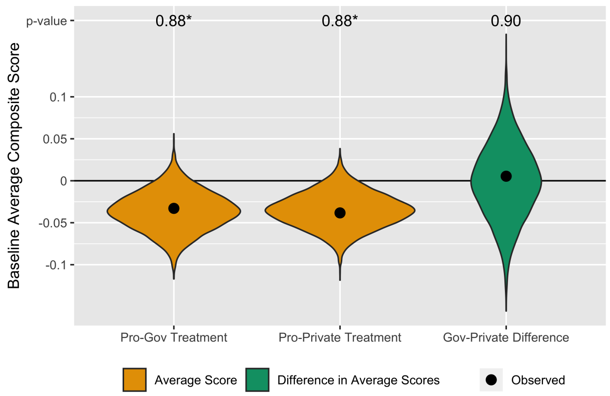

Appendix 7 Figure 1. Average Baseline Scores and Differences in Average Baseline Scores by the assigned Investment Information Group. Stars indicate that p-values are not genuine, as they depend on the calculated median of the reference distribution rather than a constant determined a priori.

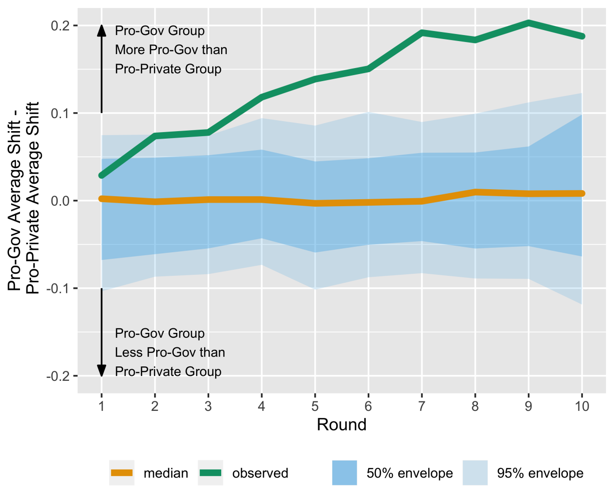

Appendix 7 Figure 2. Difference in Score Shift from Baseline Between Assigned Official Information Treatment Groups.

Appendix 8. The Marginal Effect of Social Information

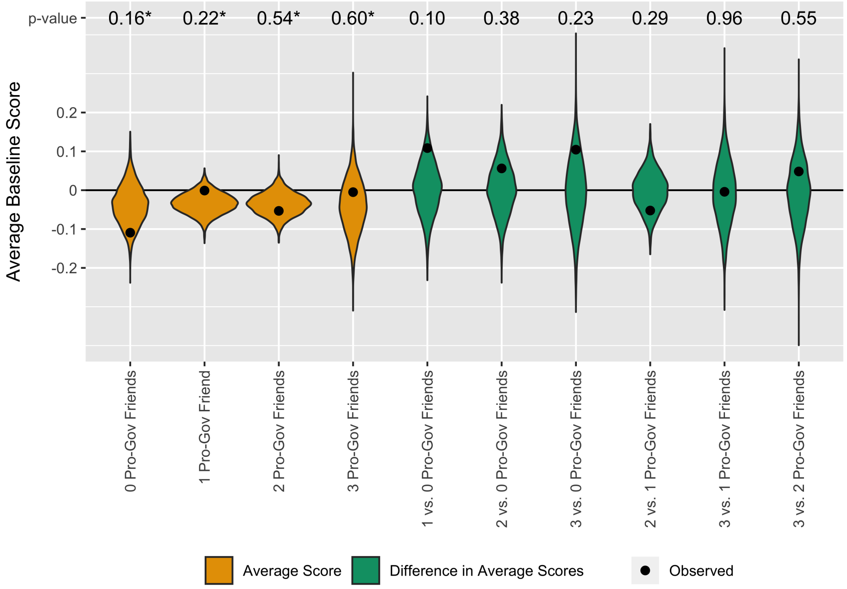

Appendix 8 Figure 1. Average Baseline Scores and Differences in Average Baseline Scores by the Number of Friends assigned to the Pro-Government Investment Information Group. Stars indicate that p-values are not genuine, as they depend on the calculated median of the reference distribution rather than a constant determined *a priori*.

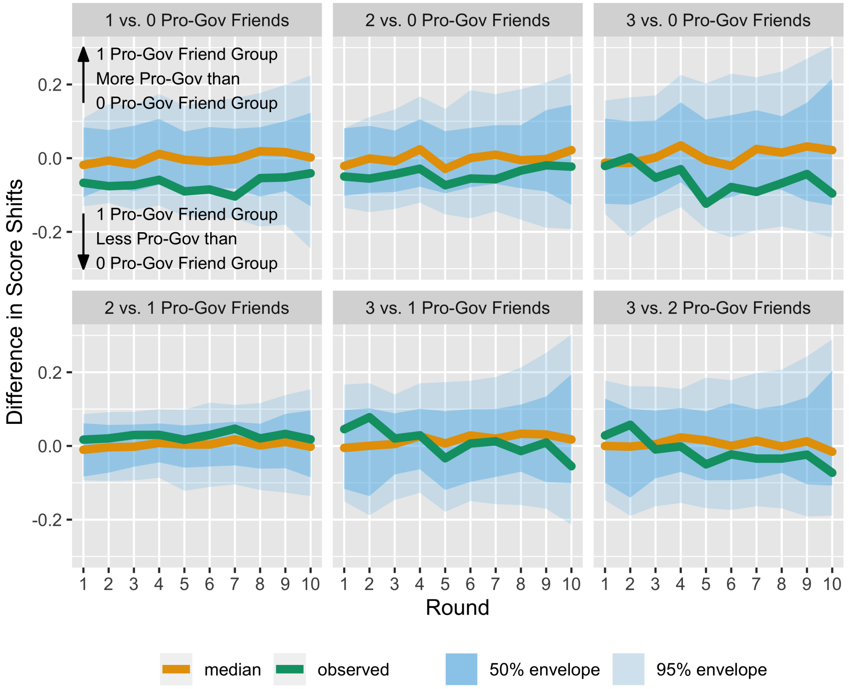

Appendix 8 Figure 2. Difference in Score Shift from Baseline Between Assigned Social Information Treatment Groups.

Appendix 9. Interaction between Official & Social Information

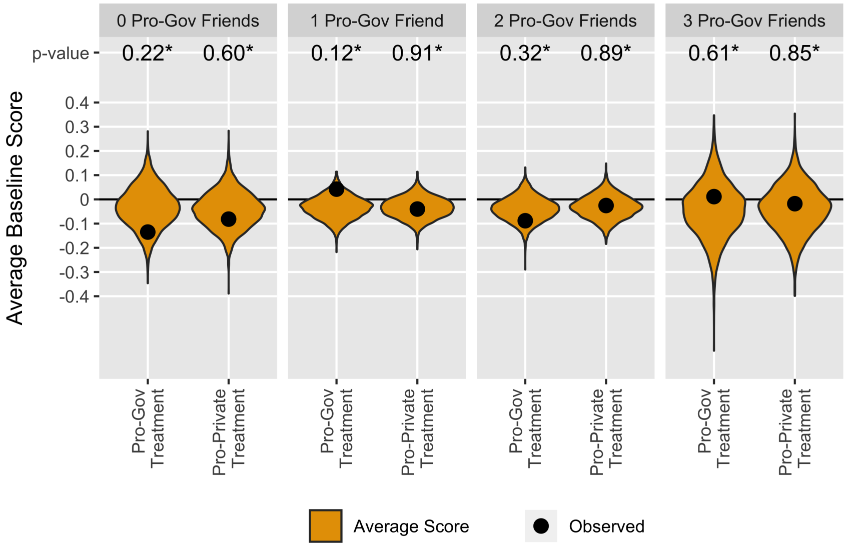

Appendix 9 Figure 1. Average Baseline Scores by the assigned Investment Information Group. Stars indicate that p-values are not genuine, as they depend on the calculated median of the reference distribution rather than a constant determined *a priori*.

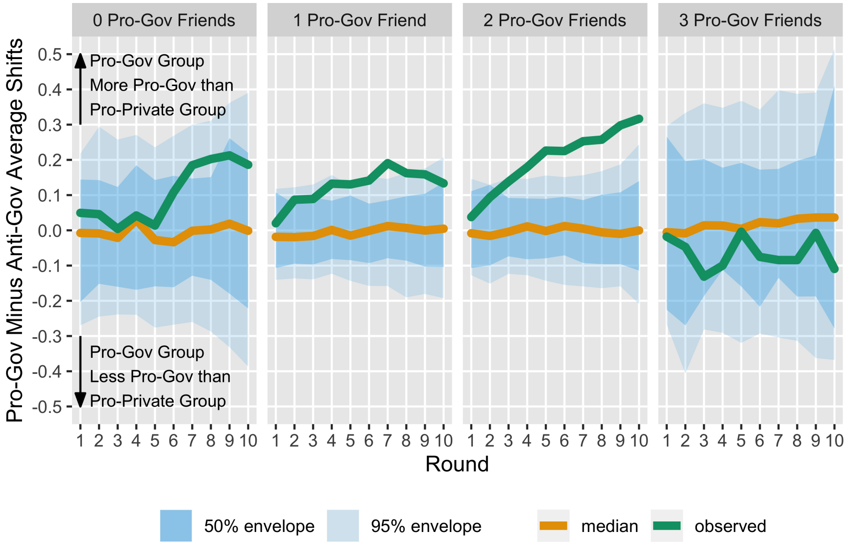

Appendix 9 Figure 2. Difference in Average Shift Across Official Information Treatment Groups, Conditional on Social Information Group.

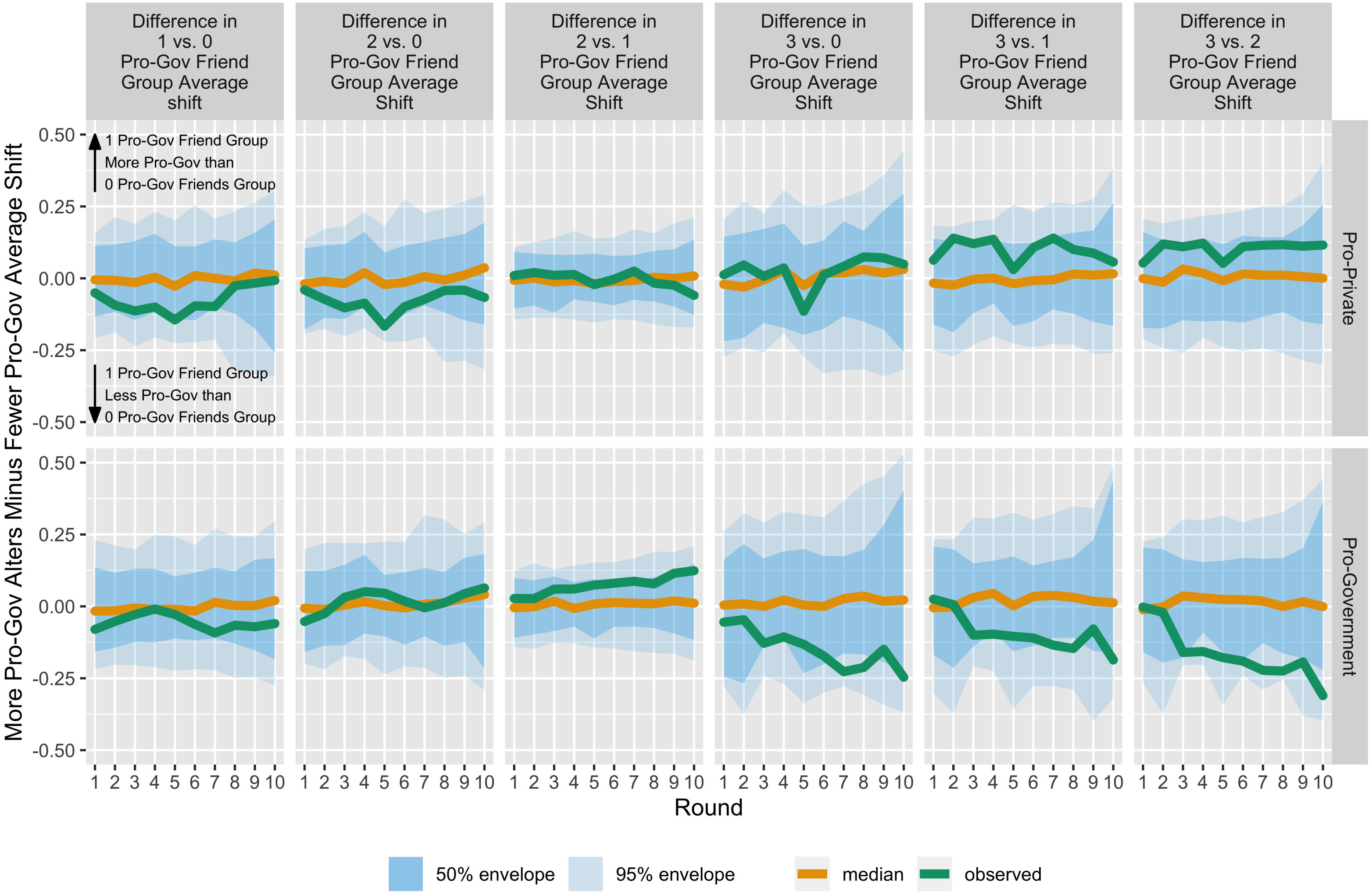

Appendix 9 Figure 3. Difference in Average Shift Across Official Information Treatment Groups, Conditional on Social Information Group.

Appendix 10. Exploring Further Effect Modifiers

It could be that learning in social networks is facilitated by *specific* social contacts and not all of them. Individuals look to their friends that are the most informed about politics when making decisions about whether to participate and which candidate to support (Huckfeldt, 2001; McClurg, 2006). In the same vein, we expect that policy information shared from the most influential friends will be heeded to a greater extent than information from less-influential peers.

We characterize these individuals as the most 'popular' group members; those group members with the largest number of people who chose them to be their friends. We call this in-degree (as out-degree is three for everyone). To do so, we looked at the number of people who chose each participant to be their friend and identified all participants with the largest in-degree as the "most popular". In some networks, there were multiple individuals with the same large in-degree. Table Appendix 10 Table 1 depicts the number of most popular friends by popular friend assignment.

	Popular Friend Assignment		
**Number of Most Popular Friends**	**ProPrivate**	**ProGov**	**NA**
0	0	0	70
1	87	67	0
2	9	3	10
3	2	0	0

Appendix 10 Table 1. Number of participants in each cross-classification defined by the number of most popular friends they chose and their popular friends' assignments.

Figure Appendix 10 Figure 1 shows the average shifts for groups based on cross-classifying the popularity and treatment assignment for a participant. Clearly the fewer "most popular" people results in broader envelopes, but all the general trends look to be in the right direction, and we really don't see much difference in eyeballing comparisons across the rows and down the columns.

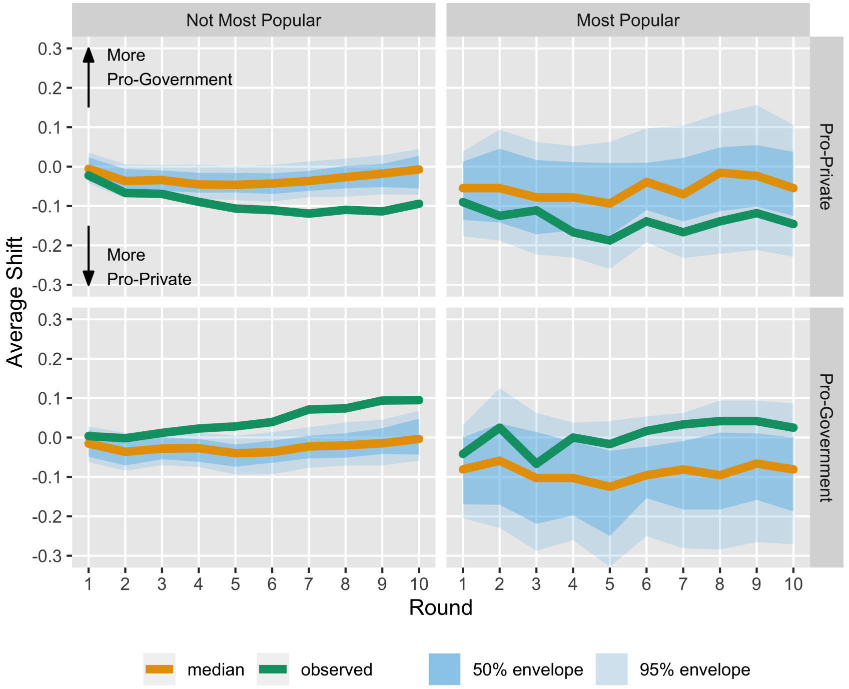

Appendix 10 Figure 1. Average Shift from Baseline by Official Treatment Group and Participant Popularity.

Figure Appendix 10 Figure 2 shows that the official information main effect trends hold steady, and there isn't much of a trend across the most popular friend(s) assignments.

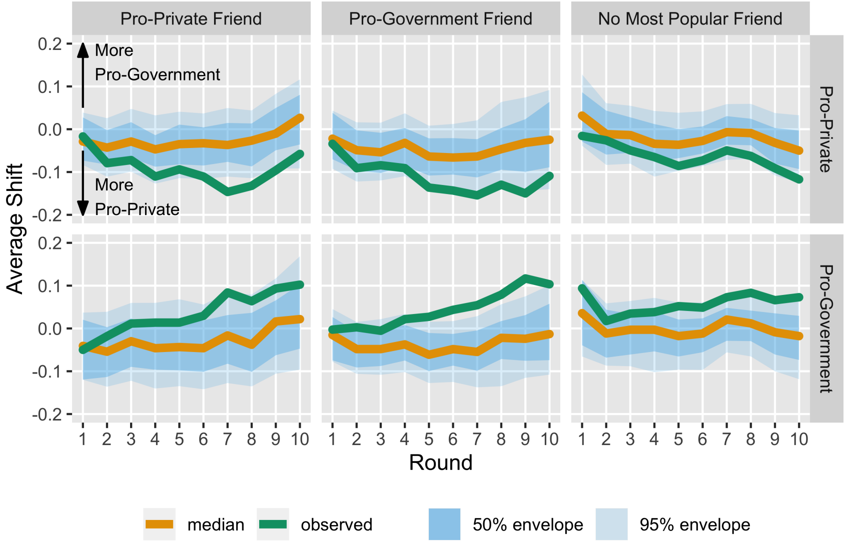

Appendix 10 Figure 2. Average Shift from Baseline by Official Treatment Group and Assignment of Popular Friends.

Taken together, we find no evidence of the effect of a popular friends' assignment on individuals'

own policy attitudes. Thus, we are more confident in our null results. Still, the social effect might operate via other channels instead, and future work should explore this more directly. We also acknowledge that our randomization works against finding an indirect effect of social information as friends display *multiple* messages, both for and against space privatization. Perhaps, if individuals were seeded the same messages from their three friends (either all pro-government or all pro-private) then we would see more of an indirect effect. This, however, would substantially complicate the design and randomization. A larger "dosage" (the number, density, or intensity) of information may be needed for social information to overcome official information effects (Neblo, Esterling, & Lazer, 2018).

Appendix 11. Statistical Analysis Details

Parametric statistical models are often used to implement or justify statistical analysis of an experiment. For the present study, we might create a parametric statistical model for the composite score outcome of interest, conditional on the treatment assignment. However, for data with a longitudinal ordinal outcome variable collected from participants connected in a network, such model-building is greatly complicated by the dependencies induced by those network connections and the discreteness of the outcome variable. We avoid the potential pitfalls of model mis-specification by using an alternative simple approach with a long history in statistical analysis of experiments, which is to use non-parametric tests of treatment effects based on permutation.

The underlying idea of permutation testing is that if the treatment had no effect on the outcomes, then we would see similar outcomes had the treatment assignments been randomized differently. That is, we can simulate a replication of the current experiment by re-doing (shuffling) the random treatment assignment without changing any of the post-randomization variable values, including the observed outcomes. Because we do not tamper with the observed outcomes or the network structure, permutation testing preserves any correlation among the outcomes that would be induced by the existing network. Thus, this type of permutation test is conditional on the network. Note that our design is completely randomized within organization (or network), and so each permutation also only reshuffles assignments within the network. For each pseudo-experiment, we calculate a summary statistic relevant to the treatment effect of interest. Repeating this procedure for many pseudo-replicate

treatment assignments creates a reference distribution of post-randomization outcome summary statistics assuming the null hypothesis that the treatment had no effect. Any truly observed summary statistic that falls in the tails of this permutation-based reference distribution indicate that the observed data would be unusual to see if the the null hypothesis were true, and thus provide strong evidence against the null hypothesis of no treatment effect. For this permutation scheme to work in the presence of missing data, we must make the permuted assignments for the whole networks (regardless of missing data) and then subset each permuted dataset after it is rejoined with the subsequent data collection to drop the appropriate participants based on their and their friends' missing data patterns. Thus, these analyses are all conditional on both the observed network structures and missing data patterns. The choice of summary statistic determines the interpretation of the test result, as well as the power to identify any true treatment effect.

This experimental setup resembles a two-factor design (see Table 1 in the main text), where the completely randomized experiment simultaneously assigns both the individual and social factor levels. The assignment of a single treatment to each participant indirectly assigns the social treatment as well, given the fixed network. As such, we choose summary statistics akin to main effects in one-way ANOVA statistical analysis or interaction effects in two-way ANOVA analyses to focus on various ways in which the treatment assignment may be associated with changes in opinion over the 10-round experiment. For each, we use the same set of 5000 permutations to create appropriate permutation-based reference distributions.

First, we examine the null hypothesis that there is no effect of treatment assignment on score shifts from baseline to round 10. For each permuted set of assignments, we calculated the average score shift among those pseudo-assigned to pro-government messages and those pseudo-assigned to pro-private messages, and then calculated the marginal effect of elite information by taking the difference between these two group-specific average shifts. Because these calculations do not involve the treatment assignments of friends, we term these measures the *marginal effect of elite information*, as they reflect the average direct treatment effect regardless of (or averaged over) friend assignments. This summary statistic is similar to the main effect on change score in a one-way ANOVA analysis. In our experimental set-up where information from treatment assignments can only be transferred to the social network via direct effects, it may be illogical to consider a case with no direct effect of treatment

assignment but with indirect effects of social information. Thus, lack of evidence against the null hypothesis would be consistent with no effect of the assigned measures, either directly or indirectly. On the other hand, strong evidence against the null hypothesis would suggest the presence of some direct effect, but would not rule out the possibility of an indirect social effect as well. As noted above, because our marginal effect of elite information calculation does not involve the network structure, the permutation distribution of this marginal measure reflects the distribution of this measure over all possible individual treatment assignments conditional on keeping the same number of each assignment within each network. That is, the permutation distribution reflects the differences in shifts that might have been observed had individual treatment assignments differed, on average across a possibly artificial distribution of indirect assignments.

To examine indirect social effects, we change from using a summary statistic focused on the direct assignment to a vector of statistics that include network structure via the number of friends assigned to view pro-government messages. For each permuted dataset, we first stratify by the number of friends who were pseudo-assigned to view pro-government messages, then calculate the pseudo-average shift from baseline within each stratum. Finally, we compare strata by subtracting the average pseudo-shift from baseline across strata for a total of 6 pairwise comparisons among the 4 strata. This procedure mirrors that used for the marginal effect of elite information, where the individual treatment assignment was used to form only two strata. As before, because we do not consider the direct individual assignment (or pseudo-assignment) for each participant in the calculated summary statistics, deviation from the reference distribution would be consistent with significant marginal effects of friend treatment assignments (i.e., social interaction) averaged over a 1:1 distribution of the direct individual treatment assignments. Because we assigned treatments to individuals via a completely randomized experiment with 1:1 randomization, the number of individuals in the strata with 0 or 3 friends assigned to view pro-government messages are expected to be much lower than the other strata. As such, estimation of effects involving these more sparse strata are likely to suffer from lower power than comparisons that involve only the more prevalent assignment strata.

Finally, we examine interactions between official and social information using the same stratification approach, but now create summary statistics based on eight individual-by-friend treatment assignment strata. Here we no longer average over one aspect of the treatment assignment to

assess marginal effects, instead choosing an analysis that more closely resembles testing for interaction effects in a two-way ANOVA analysis. Any deviation from a reference distribution comparing two strata with the same level of direct individual treatment assignment would be consistent with a *conditional* social effect within that direct assignment stratum. Likewise, any deviation from a reference distribution comparing two strata with the same level of social treatment assignment would be consistent with a *conditional* direct effect within that social assignment stratum.

Appendix 12. Social Information

Other People's Answers

Political Facts:

**Here is what your peers think:**	**Maya**	**Yael**	**Talia**
What office or political position does **Rex Tillerson** currently hold?	Secretary of Defense	Secretary of State	Secretary of State
What office or political position does **Clarence Thomas** currently hold?	Supreme Court Justice	Supreme Court Justice	Don't Know
What office or political position does **Mike Pence** currently hold?	Vice President	Vice President	Vice President
Which political party currently holds a **majority in the U.S. Senate?**	Don't Know	Republican	Republican
Which political party currently holds a **majority in the U.S. House of Representatives?**	Don't Know	Republican	Republican
**How many members** are there in the U.S. House of Representatives?	100	435	435
The United States recently announced that it would re-establish diplomatic relations with **which of the following countries?**	Russia	Cuba	North Korea

Space Policy:

**Here is what your peers think:**	**Maya**	**Yael**	**Talia**
Do you **favor or oppose** the U.S. Commercial Space Launch Competitiveness Act, which encourages private investment in space?	Favor	Strongly Oppose	Neither favor nor oppose
In your view, is it essential that the United States continue to be a world leader in space exploration over other countries like Russia and China?	Yes	No	Unsure
Would you favor the U.S. government allocating billions of dollars to send astronauts to places like the moon, Mars, and asteroids?	No	No	No
Do you think the U.S. military and intelligence agencies should setup plans for space exploration, the maintenance of strategic assets in space, and military engagement in space?	No	Unsure	No
Do you think the U.S. government should step back and rely more on private, rather than public, entities to run the country's manned space missions in the future?	Yes	No	No

Next

Appendix 12 Figure 1. This is an example of the social information each participant was shown from their three chosen friends during each communication sub-round.

References

Huckfeldt, R. (2001). The social communication of political expertise. *American Journal of Political Science*, 425–438.

McClurg, S. D. (2006). The electoral relevance of political talk: Examining disagreement and expertise effects in social networks on political participation. *American Journal of Political Science*, *50*(3), 737–754.

Neblo, M. A., Esterling, K. M., & Lazer, D. M. (2018). *Politics with the people: Building a directly representative democracy* (Vol. 555). Cambridge University Press.
